# Diagnostic accuracy and metacognition in dermatology: A cross-sectional analysis of confidence and decision-making

**DOI:** 10.1016/j.jdin.2026.01.001

**Published:** 2026-01-22

**Authors:** Matthew Helm, Angel Ray Baroz, Snehal Dhengre, Ling Rothrock, Rakefet Ackerman

**Affiliations:** a Department of Dermatology, Penn State Medical Center, Hershey, Pennsylvania; b Penn State College of Medicine, Hershey, Pennsylvania; c Penn State University, Department of Industrial and Manufacturing Engineering, State College, Pennsylvania; d Technion—Israel Institute of Technology, Department of Behavioral Sciences and Management, Haifa, Israel

**Keywords:** General Dermatology, Mental-effort regulation, Meta-Reasoning

## Abstract

**Background:**

Diagnostic accuracy in dermatology requires both visual expertise and metacognitive skills such as confidence, calibration, and decision-making under uncertainty. Miscalibration may lead to diagnostic error, unnecessary testing, and unsafe management.

**Objective:**

Assess how confidence, response time, and decision behavior vary across levels of dermatologic experience and how these factors relate to diagnostic accuracy and efficiency.

**Methods:**

This cross-sectional study included 68 participants (medical students, resident physicians, and board-certified dermatologists) who completed a multiple-choice diagnostic task with 50 diverse dermatologic images. Participants selected a diagnosis option, rated their confidence, and decided regarding additional inspection.

**Results:**

Diagnostic accuracy and confidence increased with experience. Board-certified dermatologists were most accurate when responding quickly, but not after longer deliberation, which did not hold true for residents. Medical students displayed significant overconfidence and poor alignment between confidence and decisions. Across all groups, 12% of melanomas were dismissed/overlooked.

**Limitations:**

Small sample size limits subgroup comparisons. A simulated setting may not fully capture clinical complexity.

**Conclusion:**

Metacognitive skills differ by experience and influence diagnostic accuracy and efficiency. Training should support calibration and adaptive decision-making.

**Capsule Summary**

• This study demonstrates how metacognitive skills, confidence, response time, and decision-making impact diagnostic accuracy across various training stages in dermatology.
• False confidence and miscalibration can lead to false diagnoses and inefficiencies. Incorporating metacognitive training and feedback can improve decision-making and patient safety.

## Background

Dermatology is a visual specialty where clinicians use clues such as color, morphology, and location to diagnose. Accurate image-based diagnosis in dermatology relies on domain expertise and clinicians’ metacognitive skills, which include regulation of thinking, conveying confidence, and taking effective decisions under uncertainty.^1^ Across stages of clinical training, from medical students to resident physicians to board certified dermatologists (BCDs), metacognitive skills play a pivotal role in both diagnostic success and work efficiency.^2^, ^3^, ^4^ Understanding how confidence in each diagnosis relates to success and to the invested time is critical for identifying avenues for improvement.^5^

In clinical fields, such as pathology and radiology, metacognition has been acknowledged to play a critical role in physicians’ decision-making process and exposing sources for medical errors.^6^^,^^7^ In dermatology, prior studies suggest that quick responses may reflect well-developed expertise.^8^ While aligned with this claim, our work advances the literature by empirically examining the metacognitive processes underlying such decision-making, which remain less well established.

In this pilot study, we examined how metacognitive patterns, associating confidence, response time, and decision-making with diagnosis success, differ across levels of dermatology training. Our experimental task featured real dermatology images and included participants ranging from medical students to experienced clinicians. For each case, participants selected a diagnosis, rated their confidence, and indicated the next best clinical step. By integrating dermatology-specific data with established metacognitive frameworks based on the meta-reasoning framework, we aim to provide infrastructure for inspiring training and development of automatic decision-support environments.^1^

## Methods

Sixty-seven participants were recruited through email invitations distributed by the author MH. Participation was voluntary and participants received Amazon gift cards as compensation for their time. Upon completion of the initial diagnostic exercise, participants provided information about their professional background. The cohort included 14 medical students, 31 residents, and 24 BCDs. Medical students included pre-clinical (first- and second-year) and early clinical (third- and fourth-year) learners, none of whom had completed a formalized dermatology tutorials of any sort prior, though several had exposure in dermatology through shadowing or clinical encounters. Residents were dermatology resident physicians at various stages of postgraduate training (post graduate year-2 through post graduate year-4). No minimum age requirement was used; inclusion criteria required only enrollment in medical school or residency or current certification as a dermatologist. All participants met inclusion criteria by maintaining focus on the task window, correctly answering an attention-check question, and adhering to the instructions (eg, providing varied confidence ratings).

Participants completed a 30-minute online diagnostic task after providing informed consent. The stimuli set included 50 clinical images representing diverse lesion types, sizes, and body locations. For each case, in addition to the biopsy-supported diagnosis, a dermatology expert (MH) added 2 non-trivial incorrect diagnoses to serve as distractors. Four straightforward cases were selected based on >90% accuracy in a pilot sample and represented simple, one-step recognition tasks rather than multi-step diagnostic reasoning. These cases served only to identify and exclude inattentive participants who failed to diagnose at least 2 correctly. The remaining images varied in difficulty, with success rates ranging from 7% to 88%.

All clinical photographs represented biopsy-confirmed diagnoses and were reviewed by the primary dermatology expert (MH) and 1 other BCD for quality and consistency before inclusion. Both BCDs who created the stimuli did not participate in the study as respondents. Approximately 70% of images represented lesions or growths, while 30% represented inflammatory or pigmentary conditions. No patient history or demographics (age, sex) were included to isolate image-based diagnostic reasoning and minimize bias. Dermoscopic images were intentionally excluded to align with the study’s goal of assessing metacognition under limited visual information.

**Fig 1:**
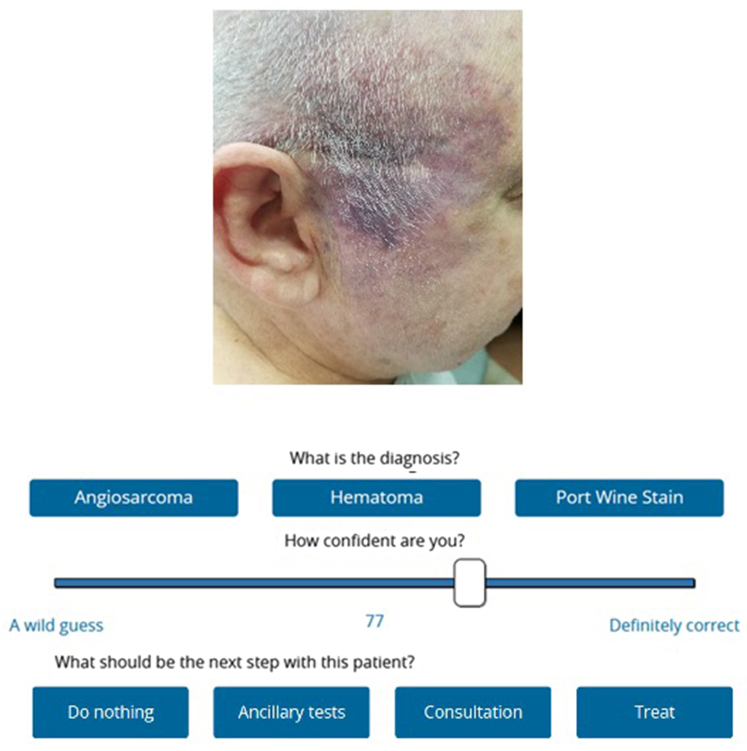
Main screen of the experimental application. Each step was sequentially activated: Choosing the diagnosis among the 3 options; when a diagnosis was clicked, the confidence rating scale appeared; and only then the next step choice was exposed.

For each case, participants chose a diagnosis from 3 options. Confidence was rated on a scale from 33% (the chance of a random guess) to 100%. After selecting the diagnosis, participants chose 1 of 4 next-step options: (1) do nothing (defined as reassurance and no intervention), (2) ancillary tests (eg, laboratory or biopsy procedures), (3) consultation (seeking a dermatology or specialty second opinion), or (4) treat (initiating a therapeutic plan). Each case was presented in random order for each participant, as illustrated in Fig 1.

## Results

**Fig 2:**
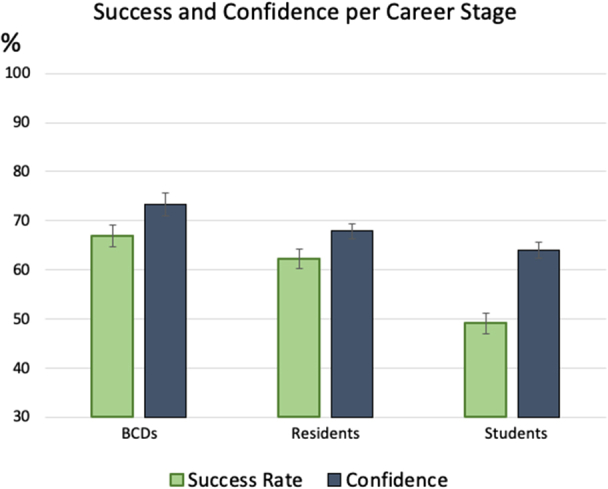
Success and confidence in division by career stage. Overconfidence is the gap between the 2 bars presented for each stage. Error bars represent standard error of the means.

Fig 2 presents the diagnostic success rate and confidence across career stages. Success rate was defined as the percentage of images correctly matched to the biopsy-supported diagnosis. Confidence was measured as the participant’s self-rated certainty (33%-100%) in their diagnostic choice. The task was challenging for all participants, and their success matched their experience level, with the medical student group lower than the other 2 stages (*P* < .001, by a Tukey post-hoc test). Success rates did not significantly differ between the resident and BCD groups (*P* = .809). Confidence ratings trended in the order of success, although the medical student group’s confidence was lower than that of the BCD group (*P* = .009) but not significantly different from residents (*P* = .358). As a result, medical students showed the largest overconfidence (see gaps between bars in Fig 2).

**Fig 3:**
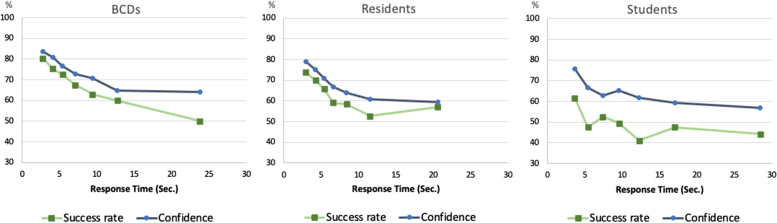
Diagnosis time course by career stage **(A)** board certified dermatologist, **(B)** residents, **(C)** medical students. The figure was drawn by splitting each participants’ response times into 7 bins. X-axis values represent the mean response time of each bin across participants. Y-axis values are means of confidence and success for each bin across participants.

Global diagnosis times did not significantly differ among the groups (*P* = .077), with the medical student group tending to the longest time. To examine time regulation in greater depth, Fig 3 presents the associations between time, confidence, and success. Overall, longer response times were associated with lower diagnostic success and lower confidence, as is well established in metacognitive research.^9^ Among BCDs, diagnostic accuracy was the best of all in their quickest responses (80%), but dropped drastically, to 50%, at the longest durations. Residents demonstrated less variability in accuracy across response times and outperformed BCDs (57% versus 50%) on cases that required more time to make a decision (average 21 seconds versus 24 seconds). Although all cases were image-only, certain images with subtle or ambiguous findings required longer deliberation, resulting in longer response times. The medical student group displayed lower success rates regardless of time investment. Their confidence ratings followed a similar trend but consistently exceeded their actual performance.

To further characterize performance differences, we calculated several composite metacognitive measures (Table I). Medical students performed the poorest across all indices, and their confidence failed to distinguish between correct and incorrect diagnoses, indicating non-significant resolution. Control sensitivity represents the within-participant correlation between confidence level and the decision to act (ancillary test, consultation, or treatment) versus to “do nothing.” This measure evaluates whether confidence appropriately guided the next-step decision. Importantly, control sensitivity does not imply that “do nothing” was incorrect; rather, it reflects how confidence influenced participants’ inclination to take or defer action. Notably, the control sensitivity measure was the only 1 that revealed a difference between BCDs and residents, with the residents not significantly different from medical students. However, only the medical student group lacked any meaningful association between confidence and their recommended action.

**Table I:** Composite measures across career stages (mean, SD)

Measure	Board-certified dermatologists	Residents	Medical students	One-Way ANOVA
Overconfidence: within-participant mean confidence minus success rate	6.3∗,^a^ (6.0)	5.5∗,^a^ (10.2)	14.8∗,^b^ (9.1)	*F*(2, 64) = 5.83, *MSE* = 78.16, *P* = .005, *η*_p_^2^ = 0.154
Efficiency (number of correct diagnoses per minute of work)	5.4^a^ (2.7)	5.1^a^ (2.3)	3.2^b^ (1.5)	*F*(2, 63) = 4.36, *MSE* = 23.79, *P* = .017, *η*^2^_*P*_ = 0.122
Resolution: within-participant gamma correlation between confidence (33%-100%) and success (yes/no)	0.35∗,^a^ (0.23)	0.23∗,^a^ (0.16)	0.07^b^ (0.20)	*F*(2, 64) = 9.17, *MSE* = 0.037, *P* < .001, *η*_p_^2^ = 0.223
Control sensitivity: within-participant gamma correlation between confidence (33%-100%) and the decision whether to do nothing or act in any of the other way	0.53∗,^a^ (0.33)	0.24∗,^b^ (0.29)	0.09^b^ (0.28)	*F*(2, 64) = 10.45, *MSE* = 0.091, *P* < .001, *η*_p_^2^ = 0.249

^a,b,c^ Different superscripts represent significant differences between career stages, *P* < .05, by a Tukey post-hoc test.

∗ Significantly larger than zero, in a one-sample t-test.

Beyond the composite measures, a potentially counterintuitive finding was the frequency of selecting active interventions rather than recommending additional information gathering. Specifically, “do nothing” was recommended more frequently among residents and medical students than among the BCDs. Similarly, medical students were more likely than others to choose treatment. A Chi-Squared test revealed a difference in distribution of next step recommendations among different levels of training (*χ*^2^(6) = 234.27, *P* < .001). Given the lower success rates, larger overconfidence, and weak resolution and control sensitivity, both findings indicate over decisiveness and reluctance to seek help among the medical student group, and, to some extent, also among residents. However, “Do nothing” may indicate a conservative orientation aligned with the principle of “first, do no harm,” especially when participants were uncertain. Therefore, some frequency of “Do nothing” may represent a cautious disposition rather than decisiveness alone.

**Fig 4:**
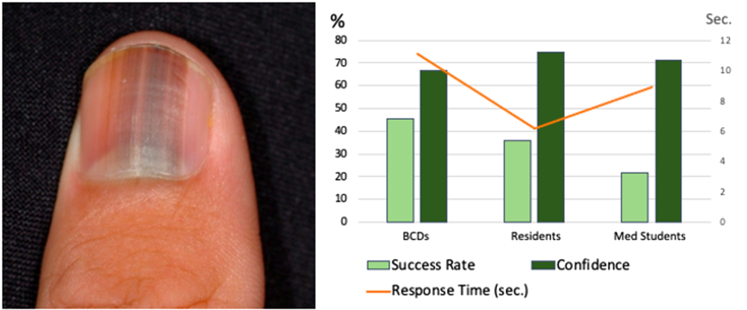
An example of a melanoma case: **(A)** Acral melanoma in situ. Lures offered: Subungual hemorrhage and longitudinal melanonychia. **(B)** Main dependent variables in division by career stages.

Overall, this in-depth analysis inspired by the metacognitive approach exposes several aspects of expertise that have not been documented so far. To illustrate our findings’ implications, we focus on the 10 melanoma cases within the study materials. The success rate was 62.8% and confidence was 69.2%. Notably, only 4 participants (6%) correctly diagnosed all melanoma cases; 2 were BCDs and 2 were residents. Fig 4 illustrates an example melanoma case, showing the mean response time, success rate, and confidence across career stages. Looking into the next step recommendations reveals that 12.1% of these melanoma cases were missed, to the extent of missing the diagnosis and recommending ‘do nothing’ as the next best step.

## Discussion

Our results expose the multi-faceted thinking process involved in dermatology. An important finding was the widespread overconfidence across all stages of training (Fig 2), aligning with the Dunning-Kruger effect.^10^ In our sample, medical students showed the poorest calibration between confidence and accuracy, meaning their self-assessed certainty often did not match their actual diagnostic success. These findings also parallel to trends in cognitive psychology, where prolonged deliberation was associated with lower success that participants often failed to recognize across all expertise levels (Fig 3).^9^ Clinicians unaware of their own uncertainty are at the greatest risk for diagnostic error, as they are less likely to seek assistance or second opinions.

Importantly, response time was a significant indicator of diagnostic quality and metacognitive control (Fig 3). A notable observation was that residents who engaged with deliberate analytical thinking could enhance performance, at least to some extent. This finding is rare in the literature. This pattern reflects prior findings where experienced clinicians are more accurate under time pressure, while those in training (residents and medical students) may benefit from slowing down and reflection.^8^^,^^11^ In terms of metacognition, the residents’ pattern of time investment reveals a strong motivation to succeed when encountering challenging cases.^12^ A relevant question for policymakers is whether residents’ slight improvement is worth the time investment it entails, or it would be better to encourage practitioners to make prompt referrals to gather additional information or consult external resources.

Confidence resolution was highest among BCDs and lowest among medical students. Confidence resolution quantifies the relationship between confidence ratings and diagnostic accuracy, indicating how well confidence differentiates correct from incorrect judgements. An important aspect that is highlighted by a metacognitive analysis is the association between resolution and control sensitivity. For effective decision making, respondents must acknowledge when they are wrong, as reflected by their monitoring (eg, confidence) resolution. Only when confidence reliably identifies the problematic cases can it effectively guide control (eg, next step) decisions that support reducing uncertainty, such as pursuing consultation or ancillary testing. Indeed, BCDs showed the best control sensitivity, meaning their decisions were more closely aligned with their confidence.^13^ The present study exposes the professional development of these metacognitive processes and their implications (Fig 4).

In contrast, medical students often chose to consult even when confident or failed to escalate care despite low confidence, as indicated by their non-significant control sensitivity. This behavioral disconnect between knowledge, confidence, and action may reflect both limited self-monitoring and external pressures, such as institutional culture or restricted autonomy in training. Supporting this interpretation, Clayton et al found that pathologists with higher metacognitive sensitivity tended to request secondary diagnostic actions, such as requesting a second opinion for inaccurate diagnoses.^14^ Notably, in our study, medical students were more likely to choose ‘do nothing’ and ‘treat’ compared to BCDs, which may reflect misjudgment of risk.

The triage of melanoma cases raised 1 of the most clinically concerning findings. While most participants correctly opted to ‘do nothing’ when faced with benign neoplasms, 12% showed failure to recognize uncertainty. This finding demonstrates the metacognitive process that leads to clinical error and mistreatment. In the era of teledermatology, where dermoscopic evaluation may be unavailable, it is important to recognize which lesions warrant in-person assessment and possible biopsy. This study underscores that even trained dermatologists may miss melanoma when relying solely on clinical images. Future studies with larger samples should aim to identify which melanoma cases are most prone to being overlooked or mismanaged in the absence of dermoscopy.

Taken together, the present findings emphasize that dermatology diagnosis requires refined metacognitive monitoring and control. Accuracy alone does not guarantee safe decision-making. Clinicians must also learn to monitor their confidence, recognize uncertainty, and appropriately regulate their actions in response. Prior research in pathology, radiology, and internal medicine has highlighted similar trends, but this study is among the first in dermatology to simultaneously evaluate accuracy, confidence, response time, and triage behavior across the full diagnostic process.^14^^,^^15^

This pilot study has several limitations. The small sample size within each subgroup limits generalizability. Participants were recruited from academic centers and volunteered to participate, introducing potential selection bias toward individuals more engaged in education or research. The diagnostic task relied on isolated clinical photographs without patient demographics, clinical history, or dermoscopic images, making it less representative or real-world evaluation. Moreover, participants came from a mix of U.S medical schools and dermatology residency programs, yet we did not collect detailed information about their training environment (such as program structure, curricular emphasis, or whether medical students were enrolled in undergraduate- or graduate-entry pathways), which restricts external generalizability. The 4 next-step decision options (“do nothing,” “ancillary tests,” “consultation,” “treat”) may have been interpreted variably among participants. Additionally, limiting each item to 3 diagnostic options may have increased chance performance and influenced confidence calibration. In future studies, expanding the number of options may better differentiate expertise and reduce guessing. Residents were at different stages of training, but subgroup analysis were limited by sample size. Finally, images were validated by 2 dermatology experts rather than a consensus panel, which could influence item difficulty calibration.

In summary, our study offers a comprehensive metacognitive tool set for delving into medical diagnosis. Clinicians across all training stages were found to be vulnerable to miscalibration and uncertainty-related errors, while demonstrating different implications by experience level. By exposing these differences, we hope to lay the ground for workflow and curricula design that support dermatologists in accurately diagnosing and reasoning carefully, under uncertainty.

## Conclusion

This pilot study provides new insights into how decision-making in dermatology is influenced by experience. While BCDs displayed the highest diagnostic accuracy when responding intuitively, their performance declined with prolonged deliberation. Residents performed mostly comparable to BCDs, but unlike them, benefited from taking additional time. Overconfidence was evident among all groups, with medical students showing the poorest calibration and weakest links between confidence, accuracy, and management decisions. These metacognitive patterns have clinical implications in patient safety and healthcare efficiency. We call for future research to develop targeted metacognitive training, focusing on self-awareness and self-regulation, as an emerging objective in dermatology education.

## Conflicts of interest

None disclosed.
